# P-1048. Coccidioides immitis Fungemia in Central California: A 10 Year Experience

**DOI:** 10.1093/ofid/ofae631.1238

**Published:** 2025-01-29

**Authors:** Sang Jo Hwang, Mohamed Fayed, Marilyn Mitchell, Geetha Sivasubramanian

**Affiliations:** UCSF Fresno, Fresno, California; UCSF Fresno, Fresno, California; Community Medical Centers, Fresno, California; UCSF Fresno, Fresno, California

## Abstract

**Background:**

Coccidioides immitis is a soil-borne fungus endemic to Southwestern United States. While about 40% of patients develop symptomatic infections, disseminated infections remain rare (1-3%). Coccidioides fungemia is extremely uncommon and associated with high mortality. Previous reports on Coccidioides fungemia in the 90’s to early 2000’s, mostly from Arizona, showed affected patients had underlying immunocompromising conditions such as HIV/AIDS, and survival was poor. We aim to characterize Coccidioides immitis fungemia in our Central California population in recent years.

**Figure:**
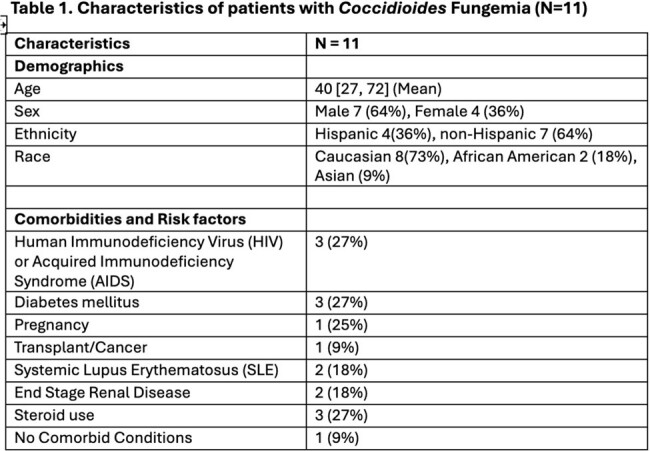

**Methods:**

We performed a retrospective analysis of patients with Coccidioides fungemia in a major 750-bed referral center in Central California between the years of 2014 – 2024. Data was collected pertaining to demographics, comorbidities, clinical presentation, management, and outcomes. Descriptive statistics were used to analyze sample characteristics.

**Figure:**
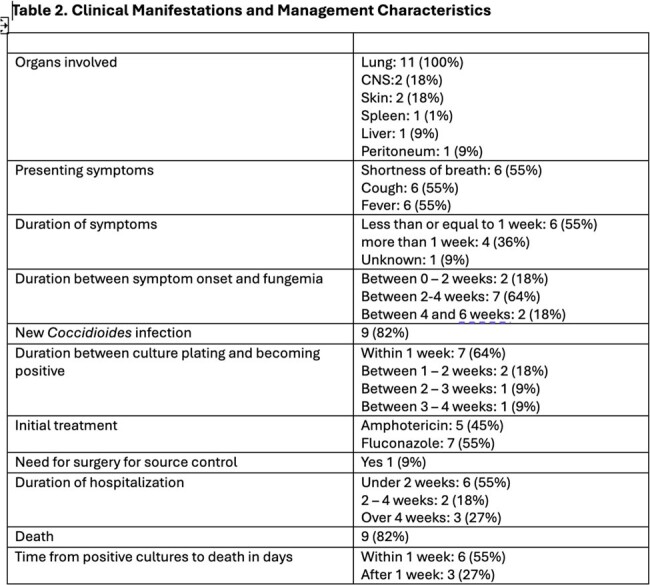

**Results:**

We identified 11 patients with Coccidioides fungemia during the study period. The mean age of affected patients was 40 years (Table 1). Mortality was extremely high with death occurring in an overwhelming 82% of patients. Duration of symptoms was less than 1 week in 6/11 (55%) of patients. Death occurred in 44%, even before blood cultures came back positive (Table 2). Cultures returned positive within 7 days of plating in 7/11(64%) patients. Notably, 9 out of 11 patients had acute Coccidioides infection. Only 1 patient had no underlying comorbid condition (Table 3). All patients received anti-fungal therapy with either liposomal amphotericin B or fluconazole.

**Figure:**
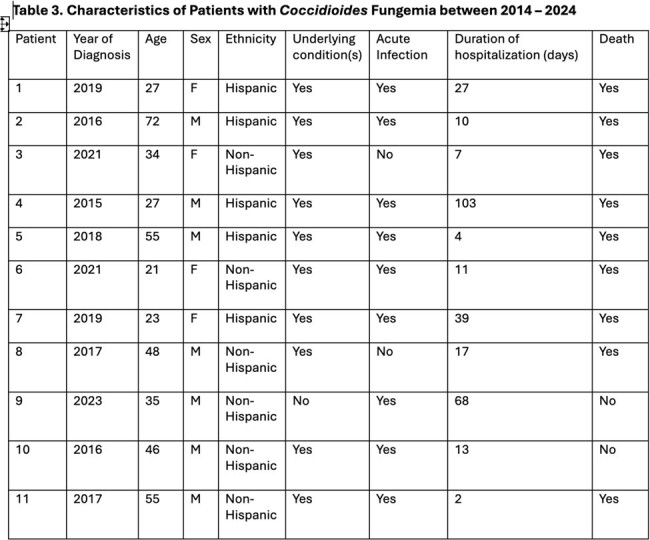

**Conclusion:**

Coccidioides immitis fungemia continues to be a rare and fatal form of disseminated disease. The majority of patients in our cohort had acute infection, rapid progression of disease and death. Only 27% of patients in our study had HIV/AIDS, which is less than what has been documented in previous studies (which were done in the early 80s-90s). However, many patients in our cohort had other underlying immunocompromising conditions such as SLE, diabetes mellitus). Despite the advances in management of HIV/AIDS, due to increase in other immunocompromising conditions and use of immunomodulatory agents, *Coccidioides* fungemia remains a threat.

**Disclosures:**

**All Authors**: No reported disclosures

